# The Father’s Part: Influences of Paternal Psychopathology and Parenting Behavior on Child and Adolescent Well-Being

**DOI:** 10.3390/healthcare11152119

**Published:** 2023-07-25

**Authors:** Stefan Mestermann, Marie Arndt, Peter A. Fasching, Matthias W. Beckmann, Oliver Kratz, Gunther H. Moll, Johannes Kornhuber, Anna Eichler, on behalf of the IMAC-Mind-Consortium

**Affiliations:** 1 Department of Child and Adolescent Mental Health, University Hospital Erlangen, Friedrich-Alexander-Universität Erlangen-Nürnberg (FAU), 91054 Erlangen, Germanyanna.eichler@uk-erlangen.de (A.E.); 2 Department of Obstetrics and Gynaecology, University Hospital Erlangen, Friedrich-Alexander-Universität Erlangen-Nürnberg (FAU), 91054 Erlangen, Germany; 3 Department of Psychiatry and Psychotherapy, University Hospital Erlangen, Friedrich-Alexander-Universität Erlangen-Nürnberg (FAU), 91054 Erlangen, Germany

**Keywords:** parenting, father, parental influence, parenting behavior, resilience, child development, parent-child-relations

## Abstract

Family influences on child quality of life (QoL) are increasingly understood. Parenting behavior and parent individual psychopathology are among the established predictors of offspring mental health. However, literature often addresses these factors as ‘parental’, lacking further gender-specific differentiation while predominantly studying maternal aspects. Social and biological fathers are still underrepresented in family research. The aim of this study was to analyze paternal contributions to child well-being. A total of 197 father/mother-dyads gave a standardized self-report on parenting behavior and their own psychopathology at child primary school age (t1; 6–10 y). Ratings were compared mutually and associated with child self-rated QoL at t1 and adolescence (t2; 12–14 y). Fathers and mothers differed in psychopathology and most parenting behavior dimensions (positive parenting, involvement, responsible parenting, poor monitoring, and corporal punishment). Father psychopathology made a relevant predictive contribution to girls’ QoL at t2. Boys’ t1 QoL was significantly influenced by maternal parenting factors (positivity and corporal punishment). Compared to mothers, fathers are faced with different individual stressors; paternal parenting behavior is different, while fathers’ influences are significant, particularly for daughters. Father-addressed pre- and intervention programs in child psychotherapeutic treatment are of high relevance.

## 1. Introduction

Bio-psycho-social factors in the developmentally relevant family–peers–school areas predict child and adolescent quality of life (QoL). Previous research provides multiple child-, parent-, and school-centered conditions with positive (protective factors) and negative (risk factors) developmental consequences [1,2]. In our study, we aimed to further specify the gender-specific influences of parental psychopathology and parenting behavior on child QoL.

Parent-related risk factors include parent mental disorders and dysfunctional parenting behavior [3]. However, literature mainly focuses on maternal reports on child development and often summarizes results as ‘parental’, without providing a gender-specific mother vs. father analysis. Maternal influences on child development and mental health are increasingly understood and are an ongoing subject of current research. Biological and social fathers (both addressed by the term ‘fathers’ in this article) are still underrepresented in family research [4,5].

Parent psychopathology tends to run through generations, is transmitted by genetic and environmental factors, has a relevant impact on parent–child interaction, and alters parenting style [6]. For example, non-responsiveness in depressive parents interferes with child emotional and social competence [7]. Similarly, verbal and physical aggressiveness in disruptive disorders, addiction, or psychosis harshly impairs child and adolescent development [8]. Further, children of parents with obsessive-compulsive disorders can experience distressful conflicts, which might impair future social competences and increase vulnerability, e.g., for anxiety disorders [9,10]. Offspring‘s social skills are decreased by histrionic, borderline, and avoidant personality disorders, even at the subclinical level [11]. Psychological distress during the COVID-19 pandemic further highlighted the correlation of parent and child mental health [12]. The mentioned studies mostly do not specify mother- or father-specific influences or, if they do, maternal data widely outweigh paternal information. Yet, differentiation is crucial, particularly because of gender-specific mental illness prevalence: women more commonly experience internalizing disorders, such as depression or anxiety, whereas men are more frequently affected by externalizing disorders such as substance use or attention deficit/hyperactivity disorders [13].

Parenting behavior is closely associated with mother/father psychopathology and has a wide impact on offspring QoL and development [14]. According to Baumrind, there are four parenting styles, depending on degree of parental demands and responsiveness towards their children: authoritative, authoritarian/controlling, permissive/indulgent, and uninvolved/neglecting [15,16]. Recent literature suggests additional dimensions; for example, parents’ disciplinary practices, parental involvement, positive reinforcement, rule setting, and extent of child autonomy [17,18]. These dimensions have a significant impact on children’s social [19] and cognitive [20] developmental outcome. Perceived parental warmth encourages the ability of long-term functional coping in future life. Authoritative parenting style predicts higher child well-being, whereas authoritarian, neglectful and hostile parents lower QoL [21]. Family support in early childhood is associated with higher QoL and daily life functionality during adulthood [22]. For German cohorts, there are no studies directly correlating parenting behavior and children’s QoL.

Parenting styles are heterogeneous and may differ between caregivers. Gender-specific influences are discussed [23]. Bem’s psychological sex role concept describes mothers as more emotion-focused and fathers as more activity- and goal-oriented [24]. Despite limited evidence, some empirical studies postulate that mothers are perceived as more supportive and responsive, but also more controlling and demanding than fathers, whereas fathers tend to show an overall more authoritarian parenting style [25]. However, paternal parenting style is a significant mediator through all stages of offspring growing-up, beginning with pre- and perinatal attachment to the child [26]. In later stages, father–child play promotes child language development compared to less involved fathers [27]. Fathers’ support and emotional sensitivity predict positive pro-social skills for adolescence and adulthood [28,29].

Study aims: Although current literature addresses parental factors, such as psychopathology and parenting styles, in child and adolescent mental health development, it often lacks mother–father differentiation. Studies mostly include mother–child dyads; fathers are still underrepresented in family research. Not only in research topics, but also in clinical practice, fathers are less involved in child therapy and are less frequently asked to participate [30], while paternal support is a crucial factor that could significantly improve therapy outcome [29,31]. The aim of this prospective longitudinal study was to analyze the gender-specific association of paternal psychopathology and parenting behavior with child QoL in childhood (t1) and adolescence (t2); with special focus on psychopathology and parenting differences between mothers versus fathers and different consequences for sons versus daughters. Thereby, we intended to clarify gender-specific family influences and define the importance of father-including therapeutic approaches in child and adolescent psychiatry.

## 2. Materials and Methods

### 2.1. Study Design and Sample Characteristics

**Figure 1:**
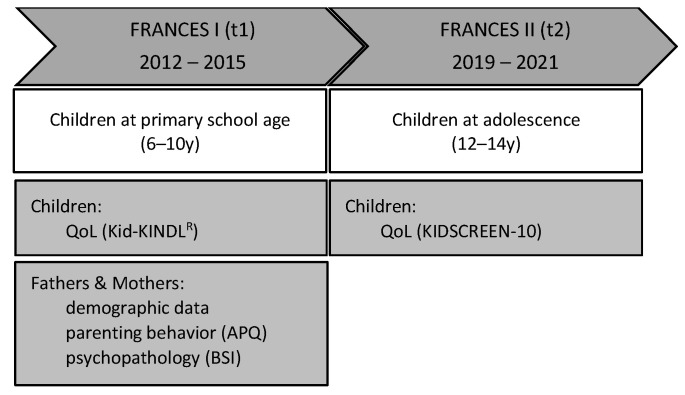
Study design of FRANCES I (t1) and FRANCES II (t2). QoL: quality of life; APQ: Alabama parenting questionnaire; and BSI: brief symptom inventory.

This study is using data from the Franconian Cognition and Emotion Studies (FRANCES) [32,33,34], a follow-up cohort study of the prospective longitudinal Franconian Maternal Health Evaluation Studies (FRAMES) [35,36]. Women were recruited at the Department of Obstetrics and Gynecology, University Hospital Erlangen (*n* = 1100) during their 3rd pregnancy trimester from 2005 to 2007 (t0). The 2nd (t1) and 3rd (t2) assessments were conducted at the Department of Child and Adolescent Mental Health. Between 2012 and 2015, when children attended primary school (age 6 to 10 years), *n* = 618 (56.2%) of these women and additionally fathers were contacted again for re-participation in the FRANCES I follow-up (t1); *n* = 245 (with *n* = 248 children) participated (39.6%; age of children: M = 7.74, SD = 0.74, range 6.00–9.90). This cohort was contacted again from 2019 to 2021, during children’s early adolescence (age 12 to 14 years), for a second data acquisition (FRANCES II, t2) [37], whereupon 76% (*n* = 186 mothers and fathers with *n* = 188 adolescents) of the FRANCES I sample re-participated (age of children: M = 13.3, SD = 0.34, and range 12.8–14.5) (see Figure 1). When comparing t2 participating families with t2 non-participating families, no differences in marital status (χ2(1) = 0.35, *p* = 0.552), family income (χ2(4) = 3.94, *p* = 0.414), or maternal total psychopathology (t(234) = −0.93, *p* = 0.353) at t1 were found. However, higher-educated parents were more often willing to re-participate (χ2(1) = 7.60, *p* = 0.006) [32].

For the present study, data of *n* = 197 mother–father–child triads during childhood (6 to 9y; t1; *n* = 98, 49.7% daughters, and *n* = 99, 50.3% sons) with additional adolescence data for *n* = 158 out of them (12–14y; t2) were available. At t1, mothers and fathers answered standardized questionnaires on their own psychopathology and parenting behavior. Children rated their QoL at t1 and t2. Mothers’ average age was 40.4 y (SD = 4.3 y), and fathers were 43.2 y (SD = 5.5 y) old. Most fathers were biologically related to child (*n* = 195; 99.0%), and 2 (1.0%) were social fathers. A total of 179 mothers (90.9%) and 186 fathers (94.4%) were of German nationality. A total of 107 mothers (54.3%) and 127 fathers (64.5%) of the cohort attended school for more than 12 years. A total of 178 women (90.4%) were in an ongoing relationship with the child’s father. Socioeconomic status (SES) was calculated based on maternal and paternal education level (4-level: <9, 9, 10, or 13 years) and net family income (6-level: <1000 to >5000) (sum index: 2 × educational level + 1 × net family income level), theoretical range: 3–14). Average SES was M = 11.43 (SD = 2.11). The study design is shown in Figure 1.

### 2.2. Measures

Parent psychopathology (t1): Mothers and fathers gave a standardized self-report about their own psychopathology at t1 via the German version of the brief symptom inventory (BSI) [38,39] (pen-and-paper format). The 53-item questionnaire asks in a 5-point Likert (0 = ‘not at all’ to 4 = ‘extremely’) format for mental stress during the last seven days, covering nine symptom dimensions (somatization, obsessive-compulsiveness, interpersonal sensitivity, depression, anxiety, hostility, phobic anxiety, paranoid ideation, and psychoticism). A summed global index (global severity index, GSI) was used in the present analyses. GSI T-values ≥ 63 indicate psychopathologic abnormality. Cronbach’s α of GSI shows very good reliability (0.93) in German cohorts [40].

Parenting behavior (t1): Mothers and fathers gave a standardized self-report about their parenting behavior via the German Version of the Alabama Parenting Questionnaire (APQ, German Version: DEAPQ-EL-GS) [41,42] (pen-and-paper format). In 72 items (5-point Likert format), parents are asked for parenting practices, resulting in seven mean scale scores: inconsistency (α = 0.72), positive parenting behavior (α = 0.84), positive involvement (α = 0.66), powerful asserting (α = 0.71), responsibility (α = 0.72), low supervision and monitoring (α = 0.75), and use of corporal punishment (α = 0.60) with a theoretical range from 1 = ‘almost never’ to 5 = ‘nearly always’.

Children’s quality of life (t1, t2): We assessed child QoL at t1 in standardized self-reports via the German version of the Kid-KINDL^R^ questionnaire [43]. Asking in 24 items on a 5-point Likert scale (1 = never to 5 = always) for aspects of child well-being during the last week, six dimensions are summed (physical well-being, emotional well-being, self-esteem, family, friends, and school). Raw sum scores are transformed into a 0–100 scale, with 100 indicating the best possible result (α = 0.85). At t2, KIDSCREEN-10 inventory was applied to quantify adolescent QoL [44]. For the past week, 10 items (‘Have you physically felt fit and well?’, ‘Have you felt full of energy?’, ‘Have you felt sad?’, ‘Have you felt lonely?’, ‘Have you had enough time for yourself?’, ‘Have you been able to do the things that you want to do in your free time?’, ‘Have your parent(s) treated you fairly?’, ‘Have you had fun with your friends?’, ‘Have you got on well at school?’, and ‘Have you been able to pay attention?’) covered the bio-psycho-social well-being of adolescents and resulted in a total value of QoL between 1 (not at all/never) and 5 (extremely/always) with α = 0.82 [45].

### 2.3. Statistical Analyses

Data were analyzed using IBM^®^ SPSS^®^ Statistics (Version 24.0). The level of significance of all analyses was defined as *p* < 0.05 (two-tailed). There were some missing ratings (QoL t1/t2 *n* = 18/3; mother/father psychopathology *n* = 5/10; and mother/father parenting behavior *n* = 3/7); therefore, analyzed groups vary from test to test. Normal (Gaussian) distribution was evaluated via Shapiro–Wilk test. Variance homogeneity was tested using the Levéne test. Mother vs. father ratings for own psychopathology and parenting behavior were compared via t-tests for independent (normal distribution) sample or Wilcoxon signed-rank test (not normal distribution), which was also used for comparison of boy’s vs. girl’s QoL ratings (t1: KID-KINDL^R^, t2: KIDSCREEN-10). T-test effect size was calculated by Cohen’s d, interpreted as 0.1–0.3 weak, 0.3–0.5 moderate, and >0.5 strong. T1 and t2 QoL ratings (total, boys, and girls) were associated with sociodemographic data and mother/father ratings for psychopathology and parenting behavior in Pearson’s (r_p_) correlations, while|r_p_| ≥ 0.10 are considered low, |r_p_| ≥ 0.30 moderate, and |r_p_| ≥ 0.50 strong/high correlation. In hierarchical linear regressions, child QoL was predicted in separate analyses for t1 and t2, and for total sample, boys and girls (resulting in six separate regression analyses). First, the significance of the parenting behavior scales and the psychopathology score for child QoL was identified in bivariate Pearson’s correlations. Second, if the bivariate correlation between the parenting behavior/psychopathology scale/score and the QoL outcome was significant, the variable was added to the regression model. In the first regression step, maternal variables were added to the model (Model 1), in the second regression step, paternal variables were added (Model 2). In some cases, there were only significant correlations for maternal or paternal predictors, resulting in only one regression step (Model 1).

## 3. Results

### 3.1. Parent Psychopathology

Regarding psychopathology, mothers reported significantly more severe symptoms (M = 47.93; SD = 13.28) than fathers (M = 44.82; SD = 12.30, *p* = 0.012) (Table 1).

### 3.2. Parenting Behavior

**Figure 2:**
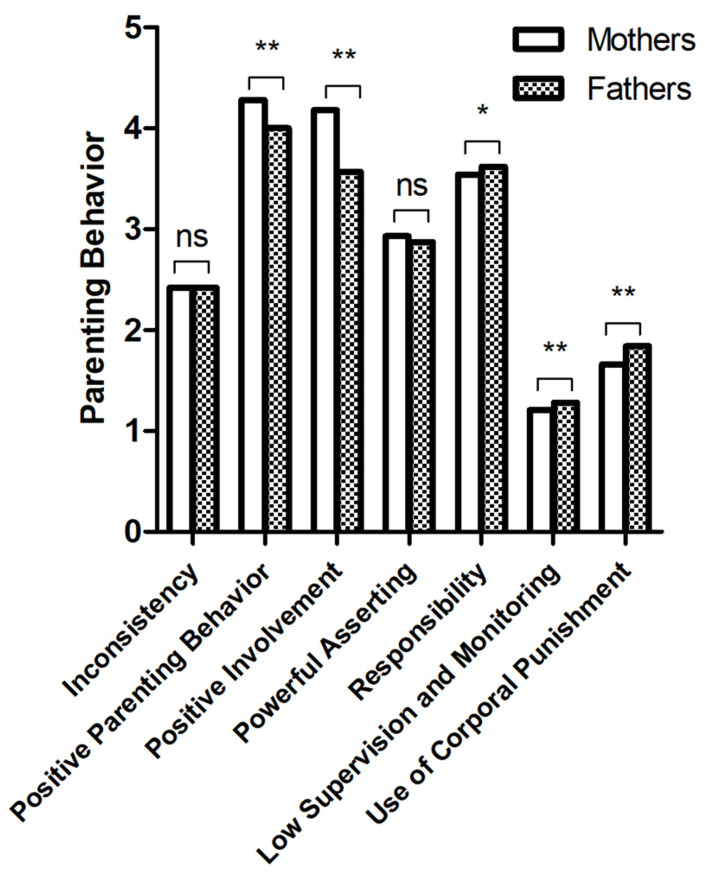
Self-reports of parenting behavior by mothers and fathers at t1 (primary school age). 1 = ‘almost never’ to 5 = ‘nearly always’; ns: not significant, * *p* ≤ 0.05; and ** *p* ≤ 0.01.

Significant differences in most parenting behavior dimensions ((*p* = 0.040 to <0.001)—with exception of ‘Powerful Assessing’ (*p* = 0.117) and ‘Inconsistency’ (*p* = 0.994)—were found (Table 1, Figure 2).

### 3.3. Child Quality of Life

At t1, there were no differences between boys and girls in their mean QoL (M = 74.83/74.68, SD = 9.45/9.15, and *p* = 0.916); the same at t2 (M = 4.24/4.18, SD = 0.42/0.54, *p* = 0.433). The overall quality of life was high in the sample (Table 2). Total primary school age QoL was weakly positively correlated with adolescence QoL (*r* = 0.29, *p* = 0.001). This was also demonstrated in gender-specific analyses for girls (*r* = 0.20, *p* = 0.092) and boys (*r* = 0.40, *p* = 0.001) (Table 2). There was no correlation of child QoL with sociodemographic factors at t1 or t2 (e.g., parent or child age or sex, parent education, and family SES) (Table 3).

### 3.4. Parent Associations with Child QoL

Correlations, to identify relevant predictors, are shown in Table 3. Regression analyses results for prediction of child (t1) and adolescent (t2) QoL by mother/father psychopathology and parenting behavior, in total and in separate for boys and girls, are figured in Table 4.

Total cohort: childhood QoL (t1): Regarding the total cohort at t1, calculations yielded significant correlations of child QoL with maternal inconsistency (*r* = −0.16, *p* < 0.05) and involvement (*r* = 0.15, *p* < 0.05), as well as with father inconsistency (*r* = −0.17, *p* < 0.05). Regression analysis revealed the strongest prediction of paternal inconsistency in parenting behavior (β = −0.14, *p* = 0.079) for child QoL. Adolescent QoL (t2): At t2, adolescent QoL correlated significantly with maternal psychopathology (*r* = −0.16, *p* < 0.05), as well as paternal psychopathology (*r* = −0.19, *p* < 0.05) and positivity (*r* = 0.18, *p* < 0.05). At t2, paternal positivity (β = 0.16, *p* = 0.055), and particularly fathers’ psychopathology (β = −0.22, *p* = 0.008), allowed the strongest prediction of adolescent QoL.

**Figure 3:**
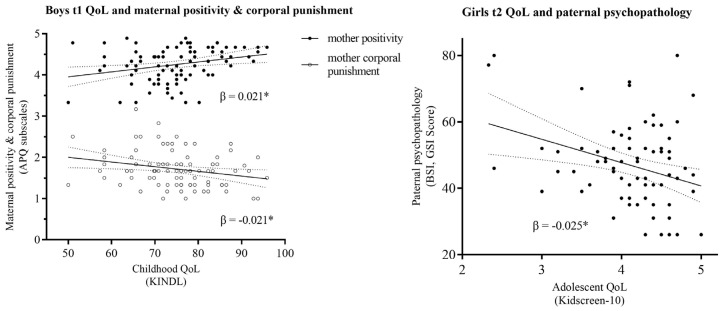
Regression models of the most significant predicting boys’ and girls’ child/adolescent QoL by parent psychopathology and parenting behavior. APQ: Alabama Parenting Questionnaire, QoL: quality of life, t1: children at primary school age (6–10 y; QoL: 0–100 via Kid-KINDL^R^), t2: children at adolescence (12–14 y), 1–5 via KIDSCREEN-10-Index), and β: standardized regression coefficient; * *p* < 0.05.

Girls: childhood QoL (t1): at primary school age (t1), gender-specific analysis for girls revealed significant correlations of father’s low monitoring (*r* = −0.23, *p* < 0.05) as well as corporal punishment (*r* = −0.23, *p* < 0.05) with girls’ QoL. Regression analysis yielded father’s low monitoring (β = −0.18, *p* = 0.099) and corporal punishment (β = −0.18, *p* = 0.106) as the strongest predicting factors (Figure 3). Adolescent QoL (t2): for adolescent girls, correlation was significant for father psychopathology (*r* = −0.25, *p* = 0.033), which also posed a significant predictor (β = −0.25, *p* = 0.033).

Boys: childhood QoL (t1): At t1, correlation of boys’ QoL was significant with maternal positivity (*r* = 0.28, *p* < 0.01), corporal punishment (*r* = −0.25, *p* < 0.05), and psychopathology (*r* = −0.27, *p* < 0.05). Regression analysis revealed both maternal positivity (β = 0.21, *p* = 0.042) and corporal punishment (β = −0.21, *p* = 0.039) as significant predictors. Maternal psychopathology also was a strong, yet not significant predictor (β = −0.20, *p* = 0.051) (Figure 3). Adolescent QoL (t2): For adolescent boys, there were significant correlations of QoL with maternal psychopathology (*r* = −0.28, *p* < 0.05) and maternal inconsistency (*r* = −0.26, *p* < 0.05). Non-significant predictors were maternal inconsistency (β = −0.19, *p* = 0.139) and psychopathology (β = −0.19, *p* = 0.142).

## 4. Discussion

This study was designed to examine paternal contributions to children’s QoL in a longitudinal childhood–adolescence mother–father–child triad cohort. We aimed to explore possible gender-specific influencing factors regarding children’s and adolescents’ mental well-being, focusing on parental psychopathology and parenting style. Mother–father differentiation in literature is lacking; particularly, fathers are less involved in family research and clinical mental disease treatment of children and adolescents. Among suspected reasons are higher maternal involvement in child care, fathers’ job duties, and thereby lack of time, and therapists’ selection bias by primarily including mothers into clinical care processes [30]. For that purpose, we intended to detect the most impactful mother- versus father-related variables, also by considering gender-specific determinants for sons’ and daughters’ QoL. As for clinical purposes, we intended to validate the possible value of father-including therapeutic approaches in systemic child and adolescent psychiatry.

Mother vs. father differences: Child QoL is widely impacted by parental mental diseases, even at the subclinical level. Father-specific data on psychopathology are lacking [46]. In our cohort, mothers reported more severe psychopathological symptoms than fathers at t1. This correlates with literature findings during all stages of offspring’s growing-up: Parenthood is more likely in women with psychological disorders than men [47,48]. Already after childbirth, mothers report psychological distress up to three times as frequently as fathers [49]. At pre-school age (3–6 y), fathers’ self-reports for psychological strain were lower than mothers’ [50]; at primary school age to adolescence, mothers of children with psychiatric diseases report higher own psychopathology than fathers; mostly depressive symptoms [51]. Although these studies support the higher psychopathology reported by mothers in our study, other questionnaires were used and did not focus on the same ages as our cohort. Further, our study did not conduct differentiation of specific psychiatric disorders in parents, but calculated an overall value of parental psychopathology. Higher rates of maternal psychopathology might be explained by onerous work–family conflicts and additional multidimensional child care demands regularly experienced by mothers [52]. Additionally, mothers report higher parenting distress than fathers [53], which might also contribute to higher ratings of psychopathology. Further, male gender stereotypes of not admitting mental distress might play a role [54].

Considering parenting behavior, mothers in our cohort reported higher levels of positive parenting behavior and positive involvement than fathers. Maternal ratings of powerful asserting, supervision and monitoring, as well as corporal punishment were lower, indicating a more authoritative parenting style of the mothers in our study. Fathers’ ratings might be interpreted as more uninvolved and/or authoritarian. Thus, we were able to replicate gender-specific differences in parenting styles [25]. Yet, influences of maternal parenting style on child QoL are better studied and verified than fathers’ [55], which causes selection bias. Further, fathers in our cohort reported a higher sense of responsibility than mothers, which implicates a child-centered care and protective stance [42] and partially contradicts literature findings of more supportive and responsible mothers [25]. Fathers’ subjective self-efficacy predicts warm father–child interaction and reciprocally causes higher parental well-being [56]. Addressing parenting behavior, contemporary changes and assimilations in parental roles must be considered [57]. This enables both parents and other caregivers to spend more quality time with their children and share educational responsibility. This was also shown in our study by high maternal and paternal ratings of positive involvement in parenting.

Gender-specific influences on child and adolescent QoL: At primary school age, maternal psychopathology, positivity, and low extend of corporal punishment were the most accurate predictors, particularly for boys’ QoL. Paternal psychopathology was the strongest predictor for QoL in adolescence, particularly for girls. At primary school age, girls’ QoL was mostly negatively predicted by paternal corporal punishment and low monitoring. This is supported by Singh et al., who found that fathers’ assistance and authoritative parenting positively influence offspring’s life satisfaction [58], but did not include gender-specific data on boys’ and girls’ outcome. Fathers’ parenting behavior was previously shown to differ towards boys and girls: fathers of girls tend to be more attentive, emotional, and sensitive [59]. Some studies postulate that daughters experience more positive parenting from mothers, too [60,61]. Our study enhances this view: There seems to be a direct correlation of child QoL and opposite-sex parenting behavior, as well as psychopathology. According to our data, girls particularly benefit from non-corporally punitive and more (functionally) supervising fathers. This is supported by family studies, where neglecting parenting was associated with dysfunctional physical and mental child development [62]. In parallel, authoritative parenting without harsh punishing and with functional monitoring enables positive child and adolescent development, which increases QoL [21]. Our data confirm that fathers’ psychopathology is another strong factor in girls’ growing up, mainly for adolescence: fathers affected by mental disorders struggle with parenting tasks, and paternal depression is a risk factor for higher rates of corporal abuse [63] and child neglect [64].

Parental psychopathology and parenting behavior are bidirectional variables that influence each other. Dysfunctional parent–child–interaction, associated with parental psychopathology and parenting style, may result in regulatory disorders during infancy [65], in emotional problems during childhood [66], and in adolescent anxiety symptoms [67] or chronic pain disorders [68]. Further, intergenerational transmission effects of parenting styles are described, indicating the offspring’s takeover of parental characteristics and thereby influencing further generation’s (e.g., grandchildren’s) QoL [69]. Consistent with our data, literature demonstrated the high influence of father–daughters and mother–sons interaction on child/adolescent mental health in a German cohort [70]. Effect sizes of regression models in our study were at most moderate, yet significant. This confirms current family research findings with similar effect sizes [71]. As the prevalence of psychological distress, QoL impairments, and mental disorders among minors increased in the last decades [29], it is crucial for clinicians in child and adolescent psychiatry and pediatrics to acknowledge the importance of parent support in mental health treatment.

Limitations and future directions: This study has several limitations. Although we used a prospective longitudinal within-subject design, data on parenting characteristics (psychopathology and parenting style) were only available at t1. Thus, long-term conclusions on influences during later growing-up are only possible to a limited extend by our data. Furthermore, additional points of data collection would be beneficial to capture a more comprehensive understanding of child development and to provide additional insights into the trajectories and stability of parenting behavior, psychopathology, and child well-being. Analysis *p*-levels were not corrected for multiple testing due to the explorative character of the study, which poses a limitation. Further, there were several dropouts at t2 (∆n = 39). Although subjective gender-specific self-reports from mothers, fathers, and children were used in our study, there were no third-party interviews included, which limits objectivity. Observational assessments of parenting behavior and standardized assessments of child well-being, in addition to self-report measures, would provide a more valid understanding of the variables under investigation. The generalizability is limited by homogenous demographic factors such as marital status and family income. Further studies should include participants with more representative characteristics. In future studies, sample size should be expanded to enhance the robustness of the present findings.

## 5. Conclusions

Mothers report higher psychological strain than fathers. Overall gender-specific QoL did not differ between girls and boys in our cohort. Our data demonstrate that fathers have an important influence on children’s well-being. Particularly for girls, fathers’ own psychopathology, low monitoring, and high level of corporal punishment increase the risk of impaired QoL. Vice versa, boys are primarily influenced by maternal parenting behavior, whereby mothers’ psychopathology, corporal punishments, and inconsistency pose risks of maladaptive child development. However, maternal positivity in parenting represents a protective factor for offspring’s QoL, especially for boys. Parent-centered interventions play a major role in child and adolescent psychiatry. Whereas mothers already regularly participate in their children’s psychotherapeutic treatment, fathers should be included more frequently in pre- and intervention programs in psychotherapeutic treatments. Future research should further investigate the distinctive roles of parents and caregivers in clinical child and adolescent mental health treatment and validate their participation in gender-specific parent interventions. Child and adolescent health specialists should keep in mind that paternal third-party anamnesis can validly contribute to mental disease diagnostics and therapy, and that where acceptable and possible, (supporting) parents and/or caregivers should be enabled to participate in treatment—fathers and mothers alike.

## Figures and Tables

**Table 1:** Self-reports of parent psychopathology and parenting behavior by mothers and fathers at t1 (primary school age).

	Mothers		Fathers				
	N	M (SD)	N	M (SD)	t (df)	|d|	*p*
Parent psychopathology (BSI)	192	47.93 (13.28)	187	44.82 (12.30)	2.53 (186)	0.18	0.012 *
Parenting behavior (APQ)							
Inconsistency	194	2.42 (0.46)	190	2.42 (0.54)	−0.01 (188)	0.00	0.994
Positive parenting behavior	194	4.28 (0.39)	191	4.00 (0.49)	6.91 (189)	0.50	0.000 **
Positive involvement	194	4.18 (0.43)	191	3.57 (0.56)	13.49 (189)	0.98	0.000 **
Powerful asserting	194	2.93 (0.47)	191	2.87 (0.48)	1.58 (189)	0.11	0.117
Responsibility	194	3.54 (0.45)	192	3.62 (0.45)	−2.07 (190)	0.15	0.040 *
Low supervision and monitoring	194	1.21 (0.26)	190	1.28 (0.32)	−2.73 (188)	0.20	0.007 **
Use of corporal punishment	194	1.66 (0.45)	191	1.84 (0.43)	−4.19 (189)	0.30	0.000 **

Notes: BSI: Brief symptom inventory, t-value ≥ 63: psychopathologic abnormality; APQ: Alabama Parenting Questionnaire; M: mean value, SD: standard deviation; * *p* ≤ 0.05; and ** *p* ≤ 0.01.

**Table 2:** Total and gender-specific HRQoL of children.

	Total		Girls		Boys				
	*n*	M (SD)	*n*	M (SD)	*n*	M (SD)	t (df)	|d|	*p*
QoL t1	180	74.76 (9.15)	89	74.68 (8.88)	91	74.83 (9.45)	−0.11 (178)	0.02	0.916
QoL t2	155	4.21 (0.48)	80	4.18 (0.54)	75	4.24 (0.42)	−0.79 (153)	0.13	0.433

Notes: QoL: quality of life t1: 0–100 (Kid-KINDLR); t2: 1–5 (KIDSCREEN-10-Index); M: mean value, SD: standard deviation, df: degrees of freedom of *t*-test, and |d|: Cohen’s d/effect size of *t*-test.

**Table 3:** Correlations of child QoL with sociodemographic and parental factors.

	HRQoL t1						HRQoL t2					
	Total		Girls		Boys		Total		Girls		Boys	
Sociodemographic:	*N*	*r*	*n*	*r*	*n*	*r*	*N*	*r*	*n*	*r*	*n*	*r*
Age child t1 [y]	180	−0.01	89	<0.001	91	−0.02	155	−0.06	80	−0.03	75	−0.10
Age father t1 [y]	170	−0.07	85	−0.15	85	0.01	147	0.03	77	0.05	70	−0.03
Age mother t1 [y]	180	−0.05	89	−0.13	91	0.02	155	−0.01	80	0.05	75	−0.13
Sex (child)	180	0.01	-	-	-	-	155	0.06	-	-	-	-
Education mother	180	0.00	89	0.04	91	−0.03	155	0.08	80	0.07	75	0.09
Education father	180	0.02	89	0.09	91	−0.06	155	−0.11	80	−0.06	75	−0.18
Family SES	180	0.06	89	0.15	91	−0.05	155	0.11	80	0.13	75	0.07
Maternal…												
Psychopathology (BSI)	175	−0.03	86	0.20 +	89	−0.27 *	150	−0.16 *	77	−0.09	73	−0.28 *
Inconsistency	177	−0.16 *	87	−0.15	90	−0.17	153	−0.10	79	0.04	74	−0.26 *
Positivity	177	0.13 +	87	−0.05	90	0.28 **	153	0.08	79	0.02	74	0.17
Involvement	177	0.15 *	87	0.09	90	0.21 +	153	0.09	79	0.03	74	0.17
Powerful assessment	177	0.11	87	0.15	90	0.08	153	0.07	79	0.04	74	0.12
Responsibility	177	0.07	87	−0.004	90	0.14	153	0.00	79	−0.10	74	0.17
Low monitoring	177	−0.05	87	−0.08	90	−0.01	153	−0.11	79	−0.11	74	−0.13
Corporal punishment	177	−0.14 +	87	−0.02	90	−0.25 *	153	−0.13	79	−0.16	74	−0.11
Paternal…												
Psychopathology (BSI)	171	−0.12	83	−0.19 +	88	−0.05	146	−0.19 *	74	−0.25 *	72	−0.09
Inconsistency	174	−0.17 *	85	−0.13	89	−0.20 +	149	−0.15 +	76	−0.10	73	−0.21 +
Positivity	175	0.10	85	0.15	90	0.05	150	0.18 *	76	0.19 +	74	0.16
Involvement	175	−0.01	85	−0.03	90	0.01	150	0.09	76	0.10	74	0.07
Powerful assessment	175	−0.01	85	0.11	90	−0.11	150	0.11	76	0.22 +	74	−0.03
Responsibility	176	0.09	86	0.10	90	0.09	151	0.15+	77	0.21 +	74	0.05
Low monitoring	174	−0.14 +	85	−0.23 *	89	−0.06	149	−0.10	76	−0.09	73	−0.11
Corporal punishment	175	−0.09	85	−0.23 *	90	0.02	150	−0.01	76	−0.03	74	0.01

Notes: HRQoL: Health-related quality of life (assessed via Kid-KINDLR at t0 and KIDSCREEN−10 at t2), t1: primary school age, t2: adolescence; parental psychopathology assessed via BSI: brief symptom inventory, parenting behavior assessed via: Alabama Parenting Questionnaire (German version: DEAPQ-EL-GS), and SES = socioeconomic status; + *p* ≤ 0.10, * *p* ≤ 0.05, and ** *p* ≤ 0.01.

**Table 4:** Regression models predicting child/adolescent QoL by parent psychopathology and parenting behavior.

		R^2^	F (df, df)	Predictors	B [95% CI]	β	*p*
T1							
Total	Model 1	0.04	3.35 (2, 171) *	Mat. Inconsistency	−2.61	−0.13 +	0.087
				Mat. Involvement	2.60	0.12	0.112
	Model 2	0.06	3.42 (3, 170) *	Mat. Inconsistency	−1.93	−0.10	0.216
				Mat. Involvement	2.63	0.12	0.106
				Pat. Inconsistency	−2.31	−0.14 +	0.079
Girls	Model 1	0.08	3.70 (2, 82) *	Pat. Low monitoring	−5.41	−0.18 +	0.099
				Pat. Corporal punishment	−3.92	−0.18	0.106
Boys	Model 1	0.16	5.56 (3, 85) **	Mat. Positivity	4.92	0.21 *	0.042
				Mat. Corporal punishment	−4.47	−0.21 *	0.039
				Mat. Psychopathology	−0.16	−0.20 +	0.051
T2							
Total	Model 1	0.02	2.93 (1, 141) +	Mat. Psychopathology	−0.01 [−0.01; 0.00]	−0.14 +	0.089
	Model 2	0.10	4.92 (3, 139) **	Mat. Psychopathology	−0.002 [−0.01; 0.00]	−0.07	0.428
				Pat. Psychopathology	−0.01 [−0.02; −0.00]	−0.22 **	0.008
				Pat. Positivity	0.16 [−0.01; 0.33]	0.16 +	0.055
Girls	Model 1	0.06	4.74 (1, 72) *	Pat. Psychopathology	−0.01 [−0.02; −0.00]	−0.25 *	0.033
Boys	Model 1	0.10	3.88 (2, 69) *	Mat. Inconsistency	−0.16 [−0.36; 0.03]	−0.19	0.139
				Mat. Psychopathology	−0.01 [−0.01; 0.00]	−0.19	0.142

Notes: F: df: degrees of freedom; B: not standardized regression coefficient (if BCa–bootstrapping was applied 95% CI is depicted); β: standardized regression coefficient; psychopathology: BSI (brief symptom inventory), + *p* < 0.10, * *p* < 0.05, and ** *p* < 0.01.

## Data Availability

The datasets generated during and/or analyzed during the current study are available from the corresponding author on reasonable request.
